# Supplementary figures and images for: Recapitulation of the accessible interface of biopsy-derived canine intestinal organoids to study epithelial-luminal interactions
Source: PLoS One. 2020 Apr 17;15(4):e0231423. doi: 10.1371/journal.pone.0231423 (PMC7164685; doi:10.1371/journal.pone.0231423)

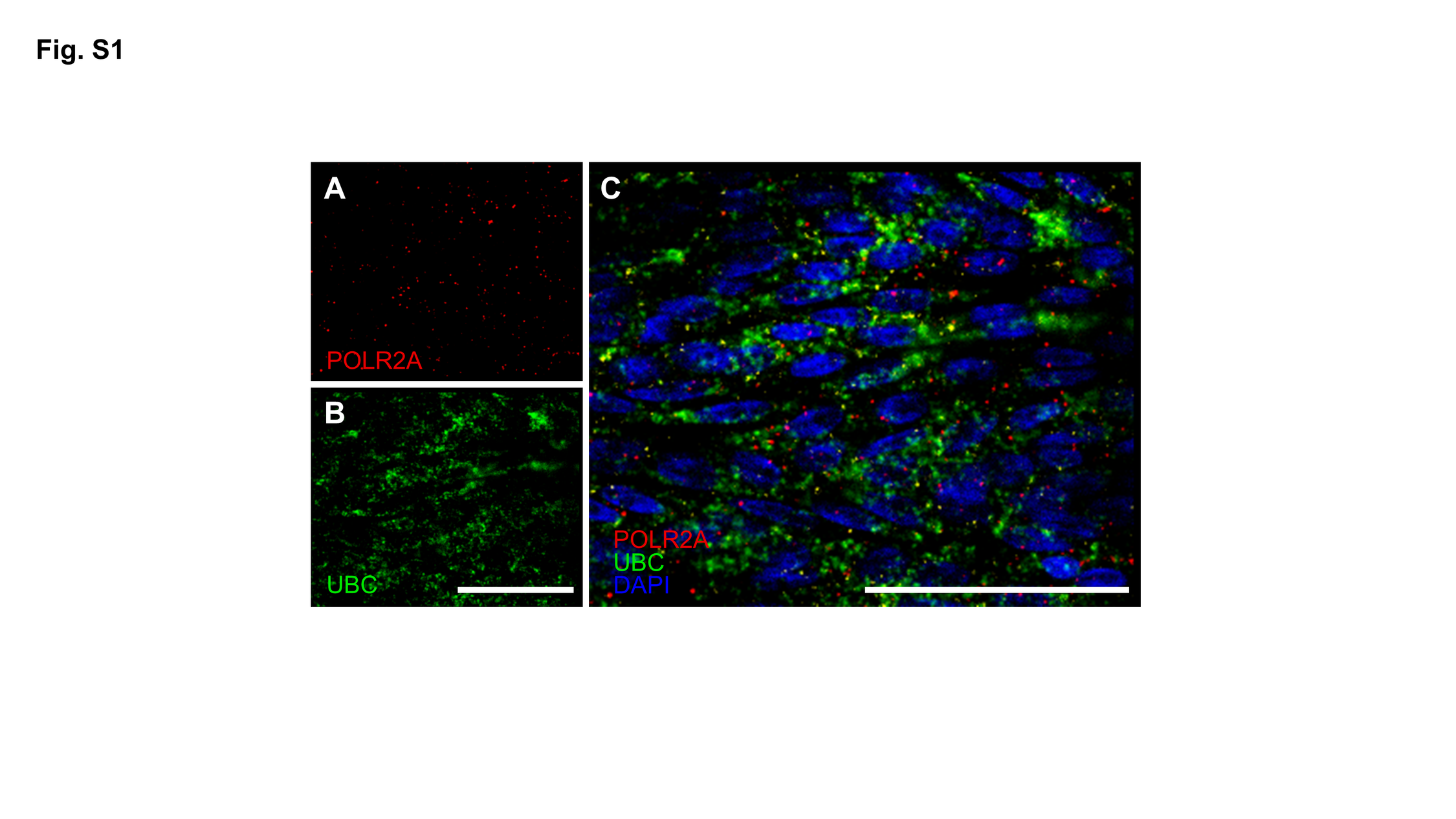

Supplement: S1 Fig — A 3-Plex Positive Control Probe (Advanced Cell Diagnostics) was applied to the canine monolayer cultured for 13 days to confirm the functionality of the kit applied. A low (RNA Polymerase II Subunit A (POLR2A), Opal 650; S1A) and a high expressor RNA (Ubiquitin C (UBC), Opal 520; S1B) confirmed the functionality of the probes applied in the canine epithelial monolayer. An overlaid image is displayed in S1C. Nuclei, blue. Bars, 50 μm. (TIF) [file pone.0231423.s001.tif]

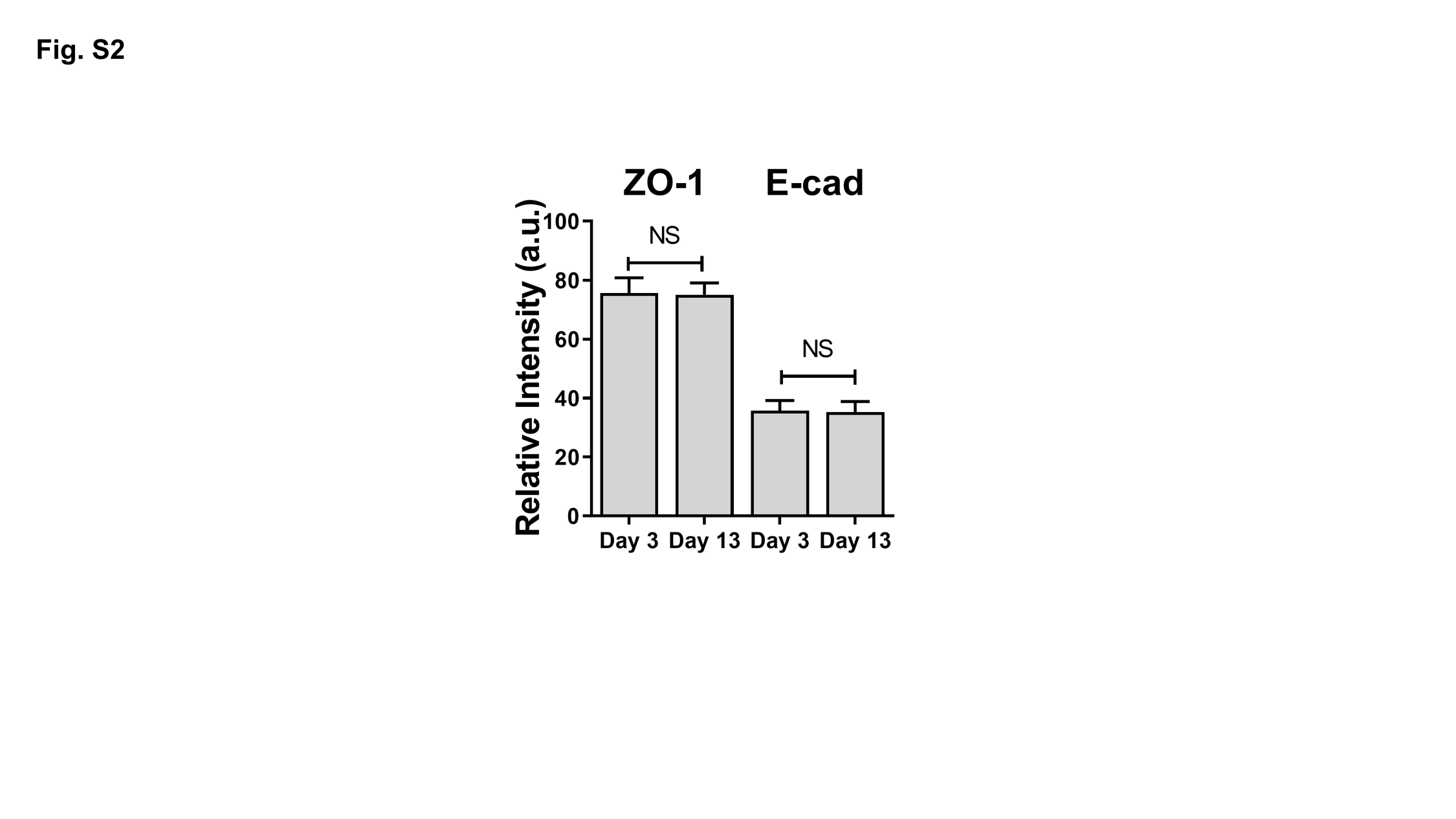

Supplement: S2 Fig — Quantification of the expression level of ZO-1 and E-cadherin at days 3 and 13 was performed using total 10 and 6 randomly chosen fields of view for ZO-1 and E-cadherin, respectively, among 4 biological replicates of IF staining experiment. We also applied two technical replicates to individual biological replicates. a.u., arbitrary unit. NS, not significant. (TIF) [file pone.0231423.s002.tif]

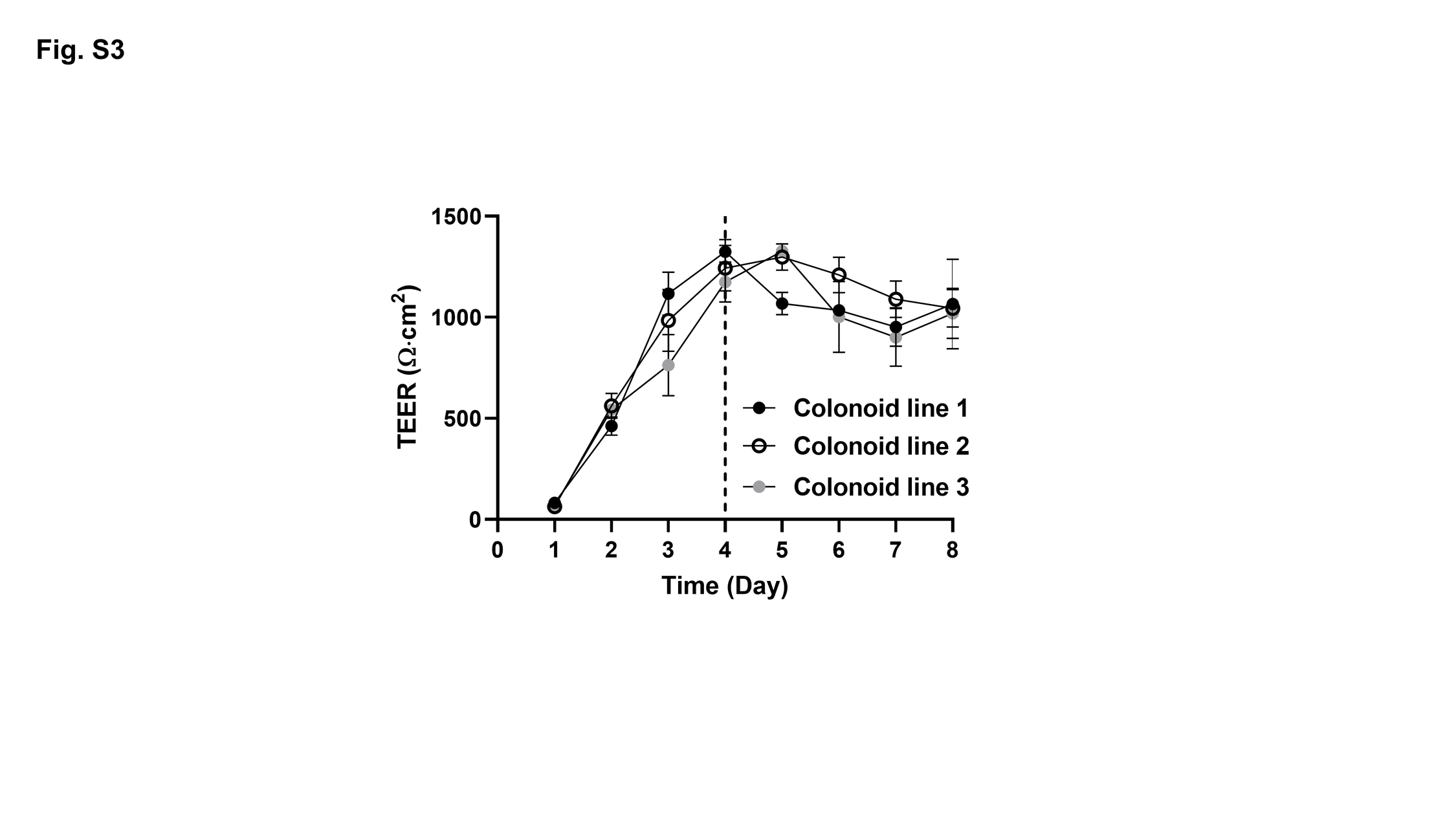

Supplement: S3 Fig — Three independent lines of canine colonoids show similar profile of epithelial barrier function when those three lines were used to form a monolayer on a nanoporous insert. The result was produced with 2 biological replicates, where each biological replicate was performed with 4 technical replicates. Error bars indicate SEM. (TIF) [file pone.0231423.s003.tif]
